# Iatrogenic Myocardial Stunning: The After-Effects of Electrical Cardioversion

**DOI:** 10.7759/cureus.81967

**Published:** 2025-04-09

**Authors:** Amit Bhandari, Soomal Rafique, Prakash Raj Oli, Avinash Murthy

**Affiliations:** 1 Internal Medicine, St. John's Hospital, Springfield, USA; 2 Internal Medicine, Southern Illinois University School of Medicine, Springfield, USA; 3 Cardiology, Prairie Heart Institute, Springfield, USA

**Keywords:** biventricular failure, cardiogenic shock, electrical cardioversion, electrical storm, myocardial stunning

## Abstract

Electrical cardioversion (ECV) is generally a safe therapy for ventricular tachycardia in unstable patients, however, associated with several complications. Potential mechanisms include myocardial stunning, rapid changes in cardiac output, and stress-induced global cardiomyopathy. We report a case of cardiogenic shock with multisystem dysfunction, likely due to myocardial stunning, in a patient who received multiple ECV treatments for electrical storm. While transient atrial stunning post-ECV is well known, severe biventricular dysfunction, though rare, is possible. This case highlights the possibility of cardiogenic shock due to myocardial stunning from ECV treatment and underscores the importance of an aggressive multidisciplinary approach to management.

## Introduction

Electrical storm (ES), characterized by less than or equal to three sustained ventricular arrhythmia episodes (including appropriate implanted cardioverter-defibrillator shocks) separated by at least 5 minutes over 24 hours [1] or incessant ventricular tachycardia (VT) lasting for >12 hours per day [2], require various medical and invasive therapies to prevent significantly high mortality [3]. Electrical cardioversion (ECV) is often required in hemodynamically unstable tachyarrhythmias [2,3]. However, complications such as transient hypotension [3], arrhythmia, thromboembolic phenomena, burns, and rarely cardiogenic shock [4,5] have been reported. With this case, we report well-known yet less frequent myocardial stunning associated with ECV.

## Case presentation

An 82-year-old man presented to an outlying hospital with shortness of breath on exertion, intermittent dull retrosternal chest pain, generalized weakness, nausea, and vomiting. His initial blood pressure (BP) was 109/72 mmHg, heart rate (HR) 140 beats per minute, respiratory rate (RR) 16 breaths/min, and oxygen saturation 96% on room air. Physical exam showed irregularly irregular tachycardia. He was started on an amiodarone drip for wide-complex tachycardia and transferred to our hospital. On arrival, he reported dizziness, nausea, weakness, diaphoresis, shortness of breath, chest pain, and palpitations. His vitals were BP 116/75 mmHg and HR 191 bpm while on amiodarone. He was immediately evaluated by the intensivist, interventional cardiology, and electrophysiology teams.

Past medical history

Our patient’s medical history included medically managed coronary artery disease, chronic diastolic congestive heart failure (Grade 2), atrial fibrillation (AFib) on apixaban, non-ischemic cardiomyopathy (NICM), dyslipidemia, diabetes mellitus type II, benign prostatic hyperplasia, Stage G3 chronic kidney disease (CKD), history of left occipital ischemic stroke, bronchial asthma, chronic allergic rhinitis, and gastroesophageal reflux disorder.

Investigations

**Figure 1:**
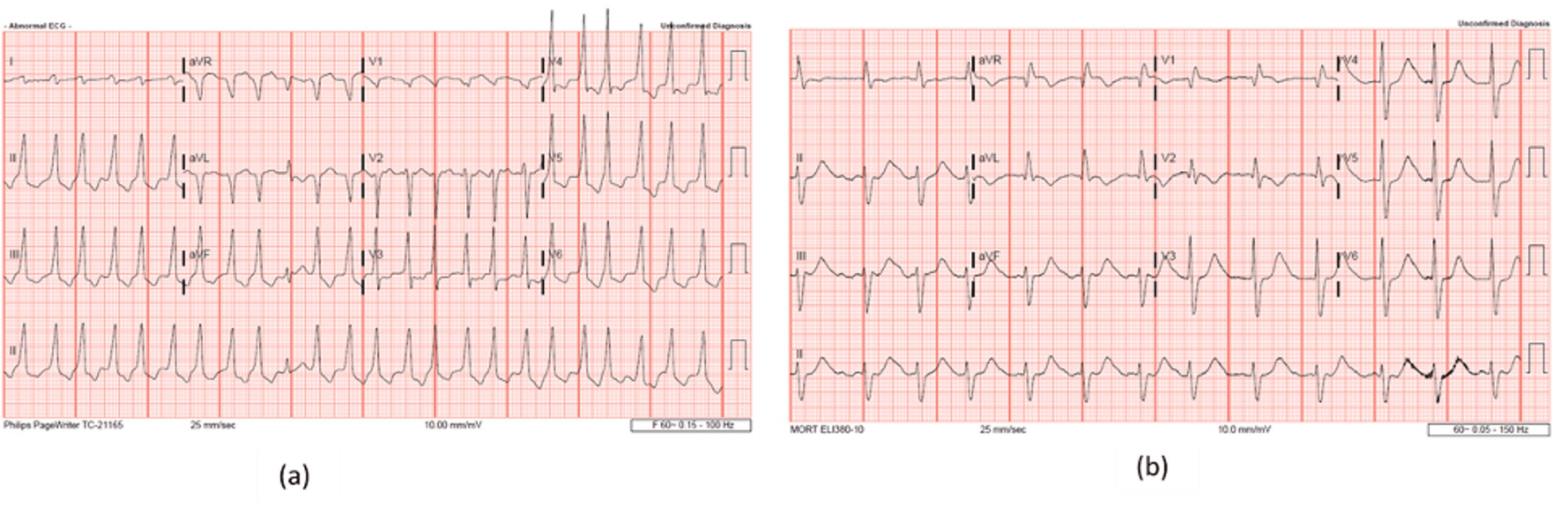
Electrocardiogram of patient done at the time of initial presentation (a) and after the VT ablation (b).

His initial electrocardiogram showed wide-complex tachycardia with a ventricular rate of 147 bpm (Figure 1a).

Chest x-ray showed no acute findings. Repeat EKG at our institute showed VT with the right bundle branch block and ventricular rate of 158 bpm. Other laboratory findings are shown in Table 1.

**Table 1:** Relevant laboratory workup.

Serum laboratory parameter	Result	Normal value
Pro-BNP (pg/mL)	4,600	<90
High sensitivity cardiac troponin I (ng/dL)	21	<12
Creatinine (mg/dL)	1.7	0.7-1.4
Blood urea nitrogen (mg/dL)	32	5-25
eGFR (mL/min/1.73m^2^)	38	>120

Management

The patient was treated with lidocaine and esmolol infusions, but his arrhythmia persisted. Due to hemodynamic instability (MAP 50, HR 190), he underwent direct current ECV with 150 J. Post-ECV, his HR stabilized, revealing underlying AFib.

**Figure 2:**
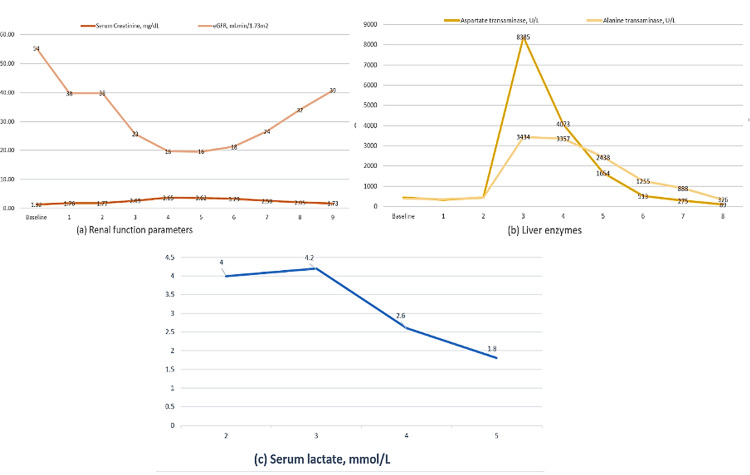
Course of the acute-on-chronic kidney injury (a), transaminitis (b) and lactic acidosis (c) over several days (as numbered on X axis).

A bedside transthoracic echocardiogram (TTE) showed left ventricular ejection fraction (LVEF) of 40%-45% with global hypokinesis. An emergent coronary angiogram showed non-obstructive coronary artery disease with moderate lesions in the proximal left anterior descending (LAD) and proximal left circumflex (LCX). Three hours later, he reverted to AFib with rapid ventricular response (HR 140) and became hypotensive. He was started on vasopressors and underwent a second ECV with 150 joules, converting to AFib with a slow ventricular response (HR 55). He was intubated and mechanically ventilated. A repeat TTE post-second ECV showed severe global hypokinesis with LVEF of 13% and moderate right ventricular systolic dysfunction with tricuspid annular plane systolic excursion (TAPSE) of 9.6 mm. Labs showed elevated lactate, liver enzymes, and worsening serum creatinine, leading to the cessation of amiodarone. A follow-up TTE the next day showed LVEF improvement to 60.2% and normal RV function (TAPSE 22.3). He remained in AFib with a slow ventricular response for over 48 hours. His vitals improved, and he was extubated on the third day. However, within eight hours post-extubation, he experienced another VT episode. He underwent radiofrequency ablation of two VT foci (LVOT and anteroseptal LV) and a PVC focus. Following ablation, he remained in AFib with controlled ventricular response (Figure 1b), stable vitals, and was weaned off vasopressors. Over the next few days, his lab parameters, including hepatic enzymes and creatinine, improved, and lactate levels normalized. Figures 2a-2c show the course of acute-on-chronic kidney injury (a), transaminitis (b), and lactic acidosis (c) from day 01 to day 09. He was subsequently discharged to a rehab facility.

## Discussion

This case report describes a case of biventricular cardiogenic shock with multisystem organ dysfunction following repeated ECV for ES/incessant VT in a patient with chronic AFib, moderate coronary artery disease, and NICM with recovered ejection fraction. The rapid recovery of biventricular function along with an aggressive multidisciplinary treatment approach led to the resolution of multiorgan dysfunction. The precise mechanisms underlying the patient’s hemodynamic collapse and cardiogenic shock are uncertain, but potential underlying processes will be discussed.

ES is a life-threatening condition characterized by less than or equal to three sustained ventricular arrhythmia episodes (including appropriate implanted cardioverter-defibrillator shocks) separated by at least five minutes over 24 hours [1] or incessant VT for >12 hours per day [2]. ES can lead to hemodynamic instability and cardiogenic shock, with short-term mortality of 10%-20% at 48 hours and long-term mortality of 20%-35% [3]. Immediate administration of various medical and invasive therapies is crucial for acute management [2]. ECV is an important and relatively safe treatment option, especially for hemodynamically unstable patients [1,2]. However, complications such as transient hypotension [3], arrhythmia, thromboembolic phenomena, burns, and rarely cardiogenic shock [4,5] have been reported. Myocardial stunning associated with ECV, rapid change in cardiac output, and stress-induced global cardiomyopathy leading to severe reduction in cardiac output have been postulated as potential mechanisms for cardiogenic shock. Most of these events were reported in patients undergoing ECV for AFib [5,6].

Risk factors for ES include cardiomyopathy, acute myocardial infarction, electrolyte imbalances, long QT syndrome, Brugada syndrome, sepsis, CKD, thyrotoxicosis, proarrhythmic drugs, digitalis use, and recent surgery [1,2,7]. Severe systolic dysfunction, CKD, and VT as an initial arrhythmia are independent predictors for ES [7]. Our patient shared many of these risk factors for ES. Managing ES is challenging, and the primary goal is hemodynamic stability and early control to prevent complications and reduce mortality [1,2]. The cornerstone of ES management includes using antiarrhythmic agents such as intravenous beta-blockers (e.g., esmolol, propranolol, sotalol) and antiarrhythmic agents such as amiodarone, lidocaine, mexiletine, and quinidine. ECV is highly effective for acutely terminating ventricular arrhythmias and is recommended for ES patients, regardless of hemodynamic stability [1,2]. Catheter ablation is recommended for incessant VT or ES caused by refractory monomorphic VT or recurrent episodes of ventricular arrhythmias from high premature ventricular contraction (PVC) burden unresponsive to medical treatment or coronary revascularization [1].

During the hospital stay, our patient experienced a sudden onset of cardiogenic shock after undergoing two ECV treatments, resulting in multiorgan failure and necessitating vasopressor support. Based on TTE findings before and after cardioversion, myocardial stunning induced by ECV was suspected to be the underlying mechanism of this acute event. Myocardial stunning is characterized by transient and reversible myocardial contractile dysfunction following acute ischemia, where blood supply is nearly restored upon reperfusion without metabolic deterioration [8]. Proposed biochemical mechanisms include the generation of oxygen free radicals leading to myocardial damage, transient calcium overload during early reperfusion, and inadequate sarcoplasmic reticulum release, resulting in excitation-contraction uncoupling [9]. Other mechanisms include insufficient mitochondrial energy production, impaired energy use by myofibrils, impaired sympathetic neural responsiveness, extracellular collagen matrix damage, and decreased myofilament sensitivity to calcium. While atrial stunning is frequently reported in patients undergoing ECV for AFib or flutter, severe biventricular failure with myocardial stunning as a contributing factor is rare [4,6]. There is no clear explanation for the cause of myocardial stunning due to ECV.

A few months before admission, our patient had a baseline LVEF of 50%-55%. After the first ECV, LVEF dropped to 40%-45% with global hypokinesis on TTE. After the second ECV, LVEF further deteriorated to 13% with severe global hypokinesis. However, within 24 hours, both the LVEF and right ventricular systolic dysfunction improved to normal levels. The rapid recovery of cardiac function and the absence of obstructive CAD on coronary angiogram support the hypothesis of myocardial stunning as the potential etiology of cardiogenic shock in our patient.

ECV-associated stress-induced Takotsubo cardiomyopathy and cardiogenic shock have been previously reported [10]. Freedman et al. reported a delayed cardiogenic shock case post-direct current cardioversion. Reviewing all cardioversion-induced shock cases until 2022, he proposed mechanisms such as post-conversion shifts in cardiac output, atrial and myocardial stunning, and stress-induced global myocardial dysfunction, often suggesting Takotsubo cardiomyopathy [5]. While it is a consideration for outpatients with cardiogenic shock, Takotsubo cardiomyopathy cannot fully explain the observed biventricular failure. Our patient had normal cardiac enzymes on presentation, and global biventricular dysfunction on TTE is unusual for Takotsubo cardiomyopathy, which is attributed to variations in catecholamine receptor localization and density [10].

Tachycardia-induced cardiomyopathy (TCM) is another potential factor for cardiogenic shock in our patient with persistent AF and presenting VT. However, the resolution of biventricular failure within 24 hours before VT ablation is atypical for TCM.

We hypothesize that the sudden alteration in the electrophysiological function of cardiac myocytes induced by ECV, potentiated by each subsequent ECV, combined with anti-arrhythmic therapy effects on cardiac electric and neuromuscular activity, contributed to myocardial stunning and subsequent cardiogenic shock. A month later, he was seen in the cardiology clinic and continued to do well, with liver enzymes and serum creatinine returning to baseline levels.

## Conclusions

ES is a life-threatening emergency requiring urgent multidisciplinary management. While ECV is generally safe, it can rarely lead to myocardial stunning and cardiogenic shock. This report describes a rare case of ECV-induced cardiogenic shock with global biventricular dysfunction, likely due to multiple factors, with myocardial stunning playing a significant role. It highlights the importance of a multidisciplinary approach in managing such complications.

## References

[1] Jentzer JC, Noseworthy PA, Kashou AH (2023). Multidisciplinary critical care management of electrical storm: JACC state-of-the-art review. J Am Coll Cardiol.

[2] Maury P, Mansourati J, Fauchier L, Waintraub X, Boveda S, Sacher F (2019). Management of sustained arrhythmias for patients with cardiogenic shock in intensive cardiac care units. Arch Cardiovasc Dis.

[3] Kern KB, Hilwig RW, Rhee KH (1996 ). Myocardial dysfunction after resuscitation from cardiac arrest: an example of global myocardial stunning. J Am Coll Cardiol.

[4] Khan MU, Khouzam RN, Khalid H, Baqir R, Moten M (2013). Cardiogenic shock following electro-cardioversion of new onset atrial flutter. Heart Lung.

[5] Freedman M, Kapoor K, Roy S (2022). Shocked into shock: Cardioversion-induced shock with multi-organ injury. Cardiol Vasc Res.

[6] Hashem AM, Al Ali O, Khalouf A (2023). Shocked to death: a case report of cardiogenic shock and death following electrocardioversion for atrial fibrillation. BMC Cardiovasc Disord.

[7] Mueller J, Chakarov I, Halbfass P (2023). Electrical storm has worse prognosis compared to sustained ventricular tachycardia after VT ablation. J Clin Med.

[8] Vaidya Y, Cavanaugh SM, Dhamoon AS (2023). Myocardial Stunning and Hibernation. StatPearls [Internet]. Treasure Island (FL): StatPearls Publishing.

[9] Bolli R (1990). Mechanism of myocardial "stunning". Circulation.

[10] Eggleton S, Mathur G, Lambros J (2008). An unusual precipitant of tako-tsubo cardiomyopathy. Heart Lung Circ.

